# Technology Development to Explore the Relationship Between Oral Health and the Oral Microbial Community

**DOI:** 10.1186/1472-6831-6-S1-S10

**Published:** 2006-06-15

**Authors:** E Michelle L Starke, James C Smoot, Laura M Smoot, Wen-Tso Liu, Darrell P Chandler, Hyun H Lee, David A Stahl

**Affiliations:** 1 Civil and Environmental Engineering, University of Washington, Seattle, WA 98195, USA; 2 Environmental Science and Engineering, National University of Singapore, 9 Engineering Drive 1, EA-07-23, Singapore 117576, Singapore; 3 Argonne National Laboratory, 9700 S. Cass Avenue, Argonne, IL 60439, USA; 4 Akonni Biosystems, Inc., 9702 Woodfield Court, New Market, MD 21774, USA; 5 Department of Bioengineering, University of Washington, Seattle, WA 98195, USA

## Abstract

The human oral cavity contains a complex microbial community that, until recently, has not been well characterized. Studies using molecular tools have begun to enumerate and quantify the species residing in various niches of the oral cavity; yet, virtually every study has revealed additional new species, and little is known about the structural dynamics of the oral microbial community or how it changes with disease. Current estimates of bacterial diversity in the oral cavity range up to 700 species, although in any single individual this number is much lower. Oral microbes are responsible for common chronic diseases and are suggested to be sentinels of systemic human diseases. Microarrays are now being used to study oral microbiota in a systematic and robust manner. Although this technology is still relatively young, improvements have been made in all aspects of the technology, including advances that provide better discrimination between perfect-match hybridizations from non-specific (and closely-related) hybridizations. This review addresses a core technology using gel-based microarrays and the initial integration of this technology into a single device needed for system-wide studies of complex microbial community structure and for the development of oral diagnostic devices.

## Introduction

Microbes comprise a major fraction of every human's biological system. They are normal residents of skin, gut, and oral/pharyngeal systems. Most often we pay little consideration to the multitudes of microbial species that inhabit our bodies. It is only when this relationship falters, resulting in adverse physiological responses such as inflammation or other disease states, that we become fully aware of their presence. Oral diseases, dental caries, and periodontitis are among the most common preventable chronic human diseases and are the result of complex microbial interactions with their environment, other microorganisms, and the host [1]. Beyond microbial pathogenesis, increasing evidence suggests that the microbial oral flora may act as sentinels of human systemic diseases such as diabetes, heart disease, low-term birth weight, and pneumonia [2-15]. A fundamental premise of the research and technology development program by our research team is that a description of the human body as a system is incomplete without an understanding of the relationship to endogenous microbiota.

Although some species are associated with oral disease (e.g. *Streptococcus mutans *is a significant contributor to caries and *Porphyromonas gingivalis *to periodontal disease), we have a remarkably incomplete understanding of diseases that may have a more complex microbial etiology. Estimates based on molecular census-taking studies suggest that the human oral cavity is home to several hundred unique microbial species [16-23]. These populations are distributed among teeth, tissue surfaces, and saliva. Remarkably, most of these microbes have yet to be brought into pure culture [16,17,24-28] – the essential prelude for characterizing their physiology and possible virulence factors. While molecular surveys have revealed much about the species that exist in the oral cavity, very little is known about the microbial community dynamics in any one individual (through time or with changing dietary and health conditions) or how the oral microbiota vary within individuals who have a specific disease or predisposition to a disease. Thus, it is essential that we develop a more comprehensive understanding of the community structure in the oral cavity, thus providing an essential foundation for the characterization of interactions among the microbial populations and their human host.

This brief review addresses the development of DNA microarray technology for rapid and reliable characterization of the oral cavity microbiota. The underlying premise is that this information will have great utility in dissecting the complex microbial etiology associated with progressive diseases (such as carries and periodontitis). The first and primary objective of the diagnostic device we are developing is to provide rapid identification and approximate quantification of key microbial populations in a small saliva specimen. Thus, the main focus of this report is to provide the conceptual and technical background for the development of a salivary diagnostics device designed to rapidly measure the microbial composition of saliva.

## The promise and practice of microarray technology

Microarrays have demonstrated utility for highly multiplexed analyses such as genome-wide expression studies, and are also increasingly applied to the study of complex microbial communities. Hundreds to thousands of target sequences (for example, corresponding to different microbial populations) can be quickly surveyed by hybridization of a small sample with a single array. There are several probe design strategies for microbial community profiling; we prefer designing probes to detect the ribosomal RNA (rRNA) with specificities to detect the common regions of sequence shared at each phylogenetic rank, and thus approximate taxonomic rank (e.g. species, genus, phylum, and domain). These phylogenetic microarrays have been employed to identify bacterial species in numerous environmental settings, including saliva [29-34]. The most widely-used microarrays for rRNA microbial identification are planar arrays printed on glass slides [29-32,34,35], although there are alternative surfaces to glass [36,37].

Our phylogenetic arrays utilize gel-pad technology, where an ordered array of small 100 μm × 100 μm × 20 μm polyacrylamide pads are photopolymerized in place before being loaded with oligonucleotide probes [38,39]. Pad dimensions can vary in size, depending on the pattern etched into a lithographic mask. The fundamental difference between gel pad arrays and other microarray surfaces is that the individual polymeric gel elements create a high density array of three-dimensional "test tubes." Probes are covalently cross-linked to the polymer backbone instead of a solid substrate, with immobilized probe concentrations capable of reaching 10 mM within individual gel elements [39]. The solution-phase nature of a gel pad microarray has a number of theoretical and practical benefits; within the context of developing an oral diagnostic, we see several major advantages to the gel-pad array platform: 1) they are reusable, thus reducing array-to-array variability and cost for the user; 2) they can directly detect the naturally amplified rRNA, alleviating bias that happens during enzymatic amplification [28,40] and thereby provide a more direct measure of target abundance; 3) they have higher probe immobilization capacity, which facilitates the detection of low abundance targets (especially in the absence of an amplification step).

The challenge of all microarray experiments, including our own, is confirming the identity of species within a sample with a high level of confidence. In order to detect non-specific hybridization, researchers typically employ the use of a mismatch probe that differs in sequence by one nucleotide relative to each perfect-match probe. Another tactic is to use multiple probes for each organism [31]. Our approach exploits the solution-phase nature of the gel elements and employs dissociation nonequilibrium analysis to resolve perfect and imperfect duplexes on the array. A reader (microscope and camera) in combination with a thermal platform is used to characterize the dissociation of the target from each probe by measuring residual signal as the temperature is incrementally increased. The resulting melting profile (signal versus temperature) is used as a measure of duplex composition (see Figure 2). This dissociation approach has been shown to help discriminate targets from closely related non-target sequences, since targets containing one or more mismatches disassociate earlier than perfect-match targets [33,39,41-45]. Discriminating perfect-match from mismatch hybridizations – a key step toward determining the presence or absence of a particular target (e.g. species) – is also influenced by such factors as the diffusivity of the gel array, the quality of the target material, and image analysis methods.

### Improving the diffusivity of gel arrays

**Figure 1:**
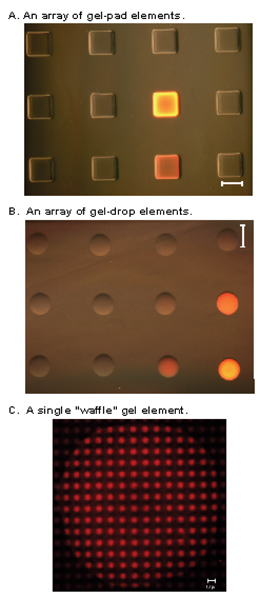
Fluorescent image of gel-pad, gel-drop, and waffle arrays. Panel A and B: fluorescent and white-light image of Alexa 594-labeled oligonucleotides hybridized to probes on a subsection of the A) gel-pad array or B) gel-drop array. Panel C: stacked confocal image of Cy3-labeled control probe on a single waffle gel element. Scale bar in Panel A and B is 100 μm and Panel C is 10 μm.

As described above, gel-pad technology has many positive attributes. However, polyacrylamide gels and the method of gel pad manufacture do impose certain limits on the otherwise solution-phase behavior of probe-target interactions. As seen in Figure 1, panel A, pads tend to generate a more intense signal on the periphery and edges of the three-dimensional gel element rather than a uniform signal throughout the gel element. The causes of this observation are multi-fold. Certainly, gel elements have a defined pore size that limits access to the gel interior; in our experience, target molecules ranging from 20–150 nucleotides are preferred and lead to the most intense and uniform signal intensity within individual pads. Secondly, the very high immobilized probe concentration has an unexpected effect on hybridization kinetics and equilibrium, eloquently described and detailed by Livshits et al. as the theory of retarded diffusion [46,47]. To illustrate, when the target first finds a probe, the target will associate with it on the most accessible part of the pad--the surface. Then by a process of repeated dissociation and reassociation with different probe molecules, the target "needles" itself into the interior of the pad. When the concentration of probe is high, this process is slower because there are more probes for the target to interact with. This process of retarded diffusion determines the time to establish equilibrium in a gel pad, where the maximum quantity of target is uniformly bound. What becomes important for microbial community profiling and unambiguously identifying perfect from imperfect duplexes on the array is that the time needed to achieve final equilibrium can be longer than the experimental hybridization conditions because of retarded diffusion. The reverse situation also applies when performing a "melt" experiment. Theory collides with practice quite quickly, since very few users of phylogenetic arrays have the luxury to wait to establish equilibrium association and dissociation conditions in order to identify perfect and imperfect duplexes. As such, we have examined the use of non-equilibrium dissociation to discriminate between perfect-match and mismatch hybridizations, and are now developing microfluidic technologies to accelerate reaction kinetics.

It is well known that active mixing or flow significantly improves microarray performance, leading to increased absolute signal intensities and lower background or non-specific binding [48-50]. Continuous washing (or flow) promotes the dissociation of all targets, but its effect is more pronounced for mismatch targets than for perfect-match targets because the dissociation rate constant of mismatch targets is higher than that of a perfect-match. Therefore, the discrimination between perfect-match and mismatch hybridizations is enhanced using a continuous flow system. To achieve this on our microarray platform, we are employing microfluidic devices, which are uniquely well suited to introducing a washing protocol. The preliminary microfluidic system requires a large buffer volume (200~400 μL) due to the fluidic lines attached to a mechanical pump. However, as technology advances, we envision embedding or integrating a small module containing a mechanical pump system to reduce the volume.

Modifications in gel element manufacturing processes may make the gel interior more accessible and "solution-like," mitigating the retarded diffusion described above. Rubina et al. [51], for example, describe a co-polymerization technique for gel element array manufacture that eliminates the photolithographic mask and associated "edges" on the gel element. Capture probes are pre-mixed with the polymer in a source plate, arrayed with conventional robotics, and photopolymerized in place, producing a "gel-drop." Since capture probes are evenly distributed throughout the gel before polymerization, they are likewise evenly distributed throughout the gel volume after polymerization [51,52] (Figure 1, panel B). In another advancement, a gel element is dissected into hundreds of micro-pillars (Selamat et al., unpublished) that improves probe distribution within the gel element (Figure 1, panel C). This "waffle-like" gel element, as compared to a normal gel element, has a three-fold increase in the effective surface area available for probe immobilization. Equal distribution of immobilized probe, however, does not eliminate retarded diffusion. Gel porosity, polymer materials, and immobilized probe concentration also have a profound effect on repeated association/dissociation throughout a three-dimensional gel element [53]. In order to counter some of these effects, several new tunable polymers are under development and testing, some of which create pores up to 300 nm, or 1/3 the size of an average bacterium. Hybridization kinetics are at least twice as rapid for 50-mer targets as the original polyacrylamide formulations used for gel pads. Several of these polymers have increased thermal stability, a property of great interest for rapid thermal melt experiments and achieving an equilibrium binding condition (during hybridization and washing) much more quickly than previously practiced. The micro-pillar modification, described above, also enhances the diffusivity of long target molecules into the gel element. This modification can increase hybridization rates and signal intensities up to five-fold compared to normal gel-elements. These improvements in hybridization kinetics can potentially enhance the accuracy of signal detection (e.g., false negative and false positive signals) during studies of microbial detection (Hong et al., unpublished). Combined with active flow from a microfluidic device, then, new developments in gel element arrays are poised to deliver on the promise of rapid analysis of microbial community composition in the oral cavity.

### Increasing signal from specimen material

Several steps in specimen preparation and processing can affect microarray results. For example, different efficiencies in the lysis of bacterial species in environmental samples can bias microarray results [34]. Toward this end, we have optimized our protocol for microbial capture and lysis from saliva samples. In addition, the length of target molecules influences the diffusivity of the material. Due to the size and the highly structured nature of 16S rRNA (nearly 1,500 bases), we fragment rRNA to ensure efficient penetration into the gel elements of the microarray. Optimization of fragmentation protocols at this step ensures quality hybridization and reproducible results [54,55].

The final step in specimen preparation is the labeling of target material with a fluorophore. Industry standards, such as Cy3, are often readily available, but as recently reported are not necessarily the best choice for experiments requiring high temperatures [56]. For example, Cy3 and Rhodamine Red lose up to 80% and 60% of fluorescence intensity, respectively, between 20°C and 80°C. Thus, for dissociation experiments using these and similar fluorophores, the decrease in signal intensity is a combination of disassociation of target and loss of fluorescence. Without quality controls, for example by adding a control probe labeled with the same fluorophore as the target in the gel array, it is difficult to deconvolute these two processes. Thus, non-temperature-dependent fluorescent dyes are preferred for signal detection at the higher temperatures used for dissociation analyses. An additional feature to consider when labeling target material is whether to use end-labeling or internal labeling strategies. Signal intensity may vary on the location of the fluorophore, particularly when it is attached at or near the ends of the target molecule [57]. Strategies that randomly label internal bases help reduce these variations in signal intensity. In addition, they have the capacity to label target molecules multiple times, which may further boost signal intensity.

### Improving image analysis

Given our need to image an array over the course of an experiment, we require software that can apply the same grid to an entire set (or stack) of images. Several artifacts such as a misaligned grid, particulate matter on the array, spot overshine, or bubble formation during an experiment can adversely affect data quality and the resulting melting profiles [58-60]. The standard image analysis tool used by our group does not retain images, given the computational and storage constraints when it was developed, and additional reanalysis is not possible [43]. In the past year, several new software programs (LabArray, AMIA, and Istackx) have been developed that allow users to extract signal intensities from images with significant quality-control measures. LabArray, which is an image acquisition and analysis tool, allows for the real-time monitoring of the probe-duplex dissociation and can instantly quantify the intensities of all spots within each image taken at a specific temperature [61]. Each image is saved and can be reanalyzed later by LabArray or other image analysis software. LabArray, developed using LabView (National Instruments, Austin TX), can simultaneously control other instrumentation components. Automated Microarray Image Analysis (AMIA) Toolbox for MatLab and Istackx are analysis tools that allow users to analyze a series of images collected by other image acquisition programs [58] (Krick et al., unpublished). AMIA provides many statistical and visual tools that enable users to quantitatively assess image analysis, including a "threading" capacity for images that are out of register (from the use of a motion controller). The Istackx program has a movie feature that displays each image in succession with the proposed grid placement to ensure the accuracy of the grid.

## Microarray interpretation

### Discrimination of perfect-match from mismatch hybridizations

**Figure 2:**
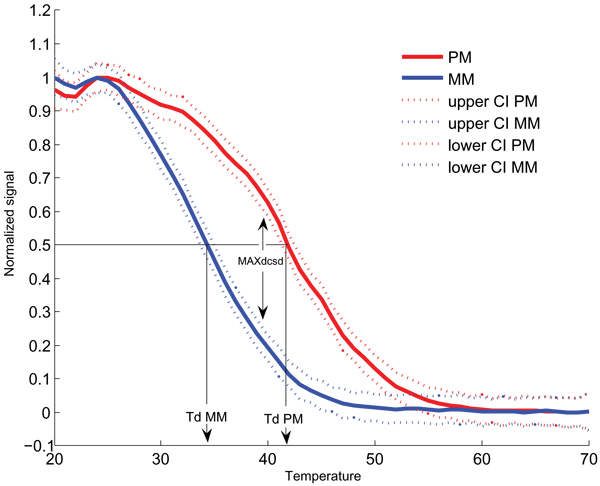
Mean melting profiles of perfect-match and mismatch probes using Alexa 594-labeled rRNA from *Streptococcus mutans *of six replicates in three experiments. Perfect-match (PM) probe and mismatch (MM) probe have T_d _(50% of signal remaining during analysis) values of 41.7°C and 34.0°C, respectively. The 95% confidence intervals are shown as dashed lines and MAX_DCSD _is 0.36 and occurs at 40°C. All values were calculated using fANOVA, see text for details (Bugli, submitted).

Once the images are analyzed and the signal intensities processed (including background subtraction and normalization), the data are interpreted to assess whether they are derived from perfect-match or mismatch targets. Evaluation of single points along dissociation curves (e.g., initial signal intensity and T_d _(the temperature at which 50% of the initial signal intensity remains)) is a useful data reduction step that simplifies data processing. The comparison of T_d _values of perfect-match probes are often greater than the T_d _of mismatch probes with the same target as expected given the greater stability of perfect-matches [43,56,62], but not all studies report effective discrimination using T_d _[42]. This result is not surprising as T_d _is influenced by many variables such as length and concentration of target, position and type of mismatch, and diffusion rates. Because the sequence and the concentration of target are unknown in oral mucosa samples, reliance on T_d _alone is problematic. Other points along the dissociation curve have been shown to have better discriminatory power than T_d_. Wick et al. describe a new metric, called T_d-w_, the temperature at which the measured *k*_d _(association constant) reaches the maxima on the dissociation rate curve [63]. Although it remains to be seen what parameters influence T_d-w_, it does out-perform T_d _in discriminating perfect-match from mismatch probes. Thus, this new parameter may be a useful tool in perfect-match/mismatch analyses. In addition, a discrimination index and neural network were used by Urakawa et al. to characterize regions of optimal discrimination between curves; however, these metrics did not provide a statistical comparison of the curves [42]. Bugli et al. developed a functional ANOVA calculator that applies statistical tools to compare differences along the entire dissociation curve and calculates a new metric, MAX_DCSD_. MAX_DCSD _is the maximum difference in normalized signal intensities between the lower limit of the 95% confidence interval of one dissociation curve and the upper limit of the 95% confidence interval of another curve (Bugli et al., submitted) (see Figure 2). In an application of the functional ANOVA calculator with nucleic acids from environmental samples, MAX_DCSD _distinguished between two curves when T_d _did not (Eyers et al., submitted), and, as with T_d-w_, the temperature at which MAX_DCSD _occurs may also be a useful parameter to monitor.

### Species Identification

Integration of the various measured parameters requires sophisticated computational procedures. For example, neural networks can process many different parameters that can define each probe-target melting profile. In a preliminary microarray study of 15,584 probe-target hybridizations with known target sequences from microorganisms found in the human oral cavity, 85% of the predicted perfect-match probe-target duplexes were identified with a neural network; however, the analysis also produced several false positive readings [59]. To highlight the utility of neural networks, the data used to test the neural network was all inclusive, demonstrating that it was able to perform reasonably well under suboptimal conditions. Another computational approach to species identification has been taken by Urisman et al. [64]. Their computational approach compares the observed signal intensities at a single temperature to the predicted energy profiles to derive a similarity score [64]. To interpret the similarity score for their data set, they developed parameters to calculate the probability of the detected species given a similarity score. Because their system has low complexity (no more than two viral species per sample), adaptation of this method to other systems, particularly those from complex communities, will require additional optimization and normalization and perhaps multiple iterations for identification. Even so, this method can be applied to all microarrays, planar and multidimensional, and it would be interesting to apply this method to hybridization results from samples taken from the oral cavity. Application of the analytical and technological advancements described here is expected to further enhance our ability to discriminate perfect-match from mismatch hybridization events. Together with the resolving power of neural networks and other computational approaches, rapid, sample-to-answer diagnostics of oral microbial communities are becoming a reality.

## Conclusion

Beyond the horizon of today's technologic sophistication lies the promises of real time monitoring of microbes in their environment and cost effective diagnostics that will allow early detection and preventive medical intervention. Previously, microarrays assisted with the rapid identification of the causative agent of SARS soon after its emergence in 2002, and microarrays are being introduced as human disease diagnostics [65,66]. Technology integration is the key to this advancement, and the economy of scale and unique physiochemical properties made possible through microfluidic technology are critical to increasing specificity and sensitivity of microarray output. Indeed, the integrated microfluidic-microarray devices we are building allow for monitoring kinetics of hybridization and dissociation within an experimental apparatus. Further, integration of specimen preparation on the same microfluidic card as the microfluidic-microarray device will release the research and clinical communities from cumbersome and laborious methodologies. As we move toward a fully integrated device, external and internal on-card quality control standards are being developed to make this device suitable for point-of-care diagnostics. There have been studies that associate the microbial response to various physiological parameters and disease development [67-69]. Ultimately, however, linking microfluidic-microarray devices with sophisticated bioinformatics will allow for longitudinal and cross-sectional studies of the microbiota and human health that until now were inconceivable. In addition to developing a more complete understanding of the relationship between endogenous microbiota and the human body; these studies will help us identify diagnostic markers for human disease (both oral and systemic) that can be used to devise effective intervention strategies.

## Competing interests

The author(s) declare that they have no competing interests.

## Authors' contributions

All authors read and approved the final manuscript.
